# Effectiveness of Frequency-Specific Microcurrent (FSM) Therapy and Relaxation in Adults with Distress: A Pilot Randomized Controlled Trial

**DOI:** 10.3390/healthcare13101151

**Published:** 2025-05-15

**Authors:** M. Graça Pereira, Ana Mónica Machado, Margarida Vilaça, Susana Faria, Isabela Monteiro, Martim Santos

**Affiliations:** 1 Psychology Research Centre (CIPsi), School of Psychology, University of Minho, 4710-057 Braga, Portugal; anamonicamachado2021@gmail.com (A.M.M.); margaridavilaca@psi.uminho.pt (M.V.); martimsantos@email.com (M.S.); 2 Centre of Mathematics (CMAT), Department of Mathematics, University of Minho, 4800-058 Guimarães, Portugal; sfaria@math.uminho.pt; 3 Portuguese Cancer League—Braga Branch, 4710-362 Braga, Portugal; isabelaperesmonteiro@gmail.com

**Keywords:** frequency-specific microcurrent therapy, relaxation, stress, emotion regulation, emotional states, emotional distress, somatic symptoms, satisfaction with life

## Abstract

**Background:** Somatic symptoms of stress are a major concern among the general population, given their severity and overall burden. **Objectives:** This pilot randomized controlled study (RCT) aimed to assess the effectiveness of frequency-specific microcurrent (FSM) therapy alone (experimental group 1 [EG1]) and combined with a relaxation intervention (experimental group 2 [EG2]), compared to a relaxation intervention alone (active control group [ACG]) and combined with placebo (passive control group [PCG]), to determine the need for a future definitive RCT. **Methods:** Participants with clinically significant levels of stress were recruited and assessed at three moments (i.e., baseline assessment [T0], at the end of six sessions [T1], and at the end of 12 sessions [T2]) on somatic symptoms and satisfaction with life (primary outcomes), perceived stress, emotional distress, emotional states, and emotion regulation (secondary outcomes). A total of 85 participants completed T0, of whom 58 were randomized into four groups. **Results:** Using linear mixed models, differences between groups revealed that the participants receiving FSM therapy reported fewer somatic symptoms and negative emotions than those who received relaxation, at T1. Within-group analysis showed that somatic symptoms and satisfaction with life significantly improved after six or twelve sessions of FSM therapy as well as after relaxation. There were also significant improvements in perceived stress, emotional distress, emotion regulation, and emotional states (negative emotions, self-efficacy, and serenity), after six and/or 12 sessions of FSM therapy or relaxation. **Conclusions:** The results suggest that FSM may be a promising treatment for addressing somatic complaints and negative emotional states, supporting the implementation of a definitive RCT.

## 1. Introduction

Somatic symptoms resulting from mental health problems are often reported as physical complaints (e.g., fatigue, weakness, pain, headache) that are not better explained by other causes, such as medical conditions or disorders [1]. Currently, it is well-established that exposure to certain potentially stressful life events or circumstances (e.g., transition to higher education, professional/academic performance difficulties) increases vulnerability to the development of clinically significant mental health problems, which, in turn, may exacerbate somatic complaints [2]. A recent study on university students found that negative emotional states and traits independently predict daily somatic symptoms, supporting the idea that these symptoms have a cognitive–affective basis and suggesting that adopting a biopsychosocial perspective is essential for a more comprehensive understanding of somatic complaints [1]. Furthermore, there is a growing trend in the prevalence of somatic complaints, underscoring the need for universal screening of somatic symptoms and mental health problems, particularly in academic populations [3]. McNealy and Lombardero [3] also emphasized the importance of distinguishing individuals with somatic symptoms from those experiencing emotional distress to better guide intervention.

A systematic review conducted before the COVID-19 pandemic, focusing on the global prevalence of adult mental health disorders, concluded that the reported increase in prevalence rates is controversial and that changes in demographic factors (e.g., age, gender, employment, level of education) may explain this increase [4]. A more recent systematic review on the prevalence of stress, anxiety, and depression among the general population found that the COVID-19 pandemic had a significant impact on individuals and community mental health, posing different physical health concerns, such as somatic symptoms [5]. The COVID-19 pandemic further exacerbated mental health challenges among adults and young adults [6], who had already been experiencing alarming levels of emotional distress [7]. A cross-sectional study conducted across multiple countries during the pandemic found that 34% of participants reported symptoms of anxiety, while 32% reported symptoms of depression [8].

Emotional distress can significantly impact the quality of life (QoL) and overall life satisfaction, interfering with daily activities, social relationships, and professional performance, leading to a reduced sense of fulfillment and decreased physical and psychological well-being [9]. Economic uncertainty, social isolation, and health concerns have been identified as significant contributing factors to increased emotional distress. Moreover, prolonged emotional distress can result in chronic stress [10,11], which subsequently leads to continuous hyperactivity of the hypothalamic–pituitary–adrenal (HPA) axis and activation of the sympathetic nervous system, thereby increasing the levels of adrenaline and cortisol [12]. The increased production of these stress-related hormones has a well-known negative impact on individuals’ lives and overall health outcomes, potentially producing and/or exacerbating the severity of somatic symptoms [10,12,13,14]. In turn, persistent and clinically significant somatic symptoms of stress (e.g., gastrointestinal disturbances, shortness of breath) have been associated with an increased risk of chronic illnesses, particularly cardiovascular diseases [15], type 2 diabetes [16], and cancer [17].

Increasing evidence also suggests that negative affectivity, both at the state and trait level, is a core mechanism underlying the development, maintenance, and course of somatic symptoms and related conditions such as stress, anxiety, and depression (e.g., [18,19]). A recent study found a strong and significant relationship between concurrent negative emotional states and somatic symptoms of burden, suggesting that negative affect modulates the processing of somatic signals [18]. Consistent with previous findings, it is expected that individuals with somatic symptoms will exhibit disturbances in the emotion regulation process, such as higher trait emotion reactivity, higher trait emotion regulation difficulties, and lower use of reappraisal strategies in daily life [20], which may contribute to lower satisfaction with life.

According to the extended process model of emotion regulation [21], emotion regulation refers to the activation of a goal to change an ongoing emotional response, and this process includes three phases: (a) identification, (b) selection, and (c) implementation. To regulate emotions, individuals need to be able to identify a situation that induces a negative emotional state and recognize the need to regulate their emotions (attention stage). However, effective emotion regulation also requires knowledge, competence, and resources (e.g., active coping), along with the ability to implement an adaptive strategy appropriate to the current situation [21]. Individuals with somatic symptoms are more vulnerable to shortcomings in this process, particularly during the attention deployment stage, which hinders their ability to identify emotions, often leading to emotional suppression and avoidance of the emotional situation [20,22,23]. If attention is not properly deployed, individuals may be unable to modify their cognitions or effectively regulate their emotional response to the situation they are facing [23]. Therefore, it is essential to identify and screen both clinical and non-clinical populations in situations of emotional vulnerability, providing them with resources and strategies to reduce emotional distress and the severity of somatic symptoms, thereby promoting greater satisfaction with life and overall well-being.

In response to the growing need for effective mental health management strategies, relaxation therapies have emerged as promising interventions, aiming to reduce the physiological and psychological symptoms associated with emotional distress [24]. Relaxation techniques are effective in promoting both physical and psychological well-being by regulating breathing, lowering blood pressure, reducing stress, and alleviating muscle tension [25,26]. Furthermore, recent research has emphasized the potential of relaxation techniques as part of a multidisciplinary approach to prevent or address stress, anxiety, and depression [27,28]. According to a recent review article, Schultz’s autogenic training (AT), one of the well-known self-induced relaxation techniques, is effective in improving mood, anxiety, stress-related disorders, and other mental health issues such as functional sleep disorders [29]. AT has also shown promising results in the management of somatic complaints, such as pain, gastrointestinal problems, fatigue, and headaches [30]. In a progressive learning logic, Schultz’s AT comprises six types of exercises (i.e., heaviness, warmth, calm heart, breathing, stomach, and cool forehead) designed to facilitate passive concentration on bodily sensations. The goal is to regulate the heart and/or respiratory rate, promoting an overall sense of well-being [31].

In addition to relaxation therapies, such emerging treatments as frequency-specific microcurrent (FSM) therapy have also shown promising results in addressing emotional distress [32] and relieving somatic issues [33]. FSM uses paired frequencies delivered through low-intensity microampere currents, grounded in the principles of biological resonance [33,34]. This therapeutic modality is performed using a microcurrent device capable of delivering adjustable frequency pairs tailored to the resonant properties of specific tissues and organs, thereby helping to modulate pain and symptoms associated with specific clinical conditions [35]. For instance, FSM has been shown to modulate somatic symptoms by influencing cellular signaling pathways and reducing inflammatory responses, thereby facilitating tissue repair and contributing to the relief of distinct somatic complaints (e.g., pain, fatigue) [34]. Several studies have investigated microcurrent therapy as a non-invasive approach with significant effects in the treatment of insomnia and sleep disorders [36], memory impairment and neuroinflammation [37], pain management (e.g., [38]), and emotional distress (e.g., anxiety, depression) [39,40,41,42]. In contrast, a study by Stößlein and Kuypers [43] found no effects of FSM on emotional states. Despite this, growing evidence supports that FSM is a safe, non-painful, and non-invasive intervention [35,37,44] that may serve as a complementary treatment for stress, anxiety, and depression. A pilot study conducted by Duke et al. [45] found that 10 and 20 sessions of microcurrent neurofeedback had a significant positive effect on symptoms of depression and anxiety. Another recent study [46] showed significant and clinically relevant improvements in health-related QoL in patients with depression using a microcurrent portable device. Despite recent efforts to demonstrate the multiple benefits of FSM on patients’ health outcomes, clinical evidence regarding the effectiveness of FSM therapy in distressed adults remains limited. Previous studies have also emphasized the importance of investigating the potential of FSM in combination with other therapies (e.g., [35]), and, to the best of our knowledge, no studies have examined the efficacy of FSM when combined with relaxation. Given the aforementioned gaps, which highlight the scarcity of clinical research on the impact of FSM on somatic symptoms, and the pressing need for more empirical data to determine the effects and specific mechanisms of FSM, particularly on emotional processes, the present study included a randomized controlled trial (RCT). The goal was to contribute to the evaluation of FSM therapy efficacy, alone and in combination with a relaxation intervention, on the reduction of somatic symptoms and overall satisfaction with life in adults with clinical stress levels. The specific aims of this study were as follows: (1) to compare the effects of FSM therapy (experimental group 1—EG1) and FSM therapy combined with a relaxation intervention (experimental group 2—EG2) with relaxation alone (active control group—ACG) and relaxation combined with placebo FSM (passive control group—PCG) over time, on somatic symptoms, satisfaction with life, perceived stress, emotional distress, emotion regulation, and emotional states and (2) to analyze differences within groups, on somatic symptoms, satisfaction with life, perceived stress, emotional distress, emotion regulation, and emotional states, over time. This study may help inform future public health policies and serve as an empirical basis for further intervention programs focused on promoting emotion regulation and physical/psychological well-being.

## 2. Materials and Methods

### 2.1. Trial Design

This study was a longitudinal, parallel, pilot RCT with a 1:1:1:1 allocation ratio. The participants were assessed at three moments: pre-test (T0), during the intervention (after the 6th session) (T1), and at the end of the intervention (after the 12th session; T2). The study protocol was registered on ClinicalTrials.gov (identifier: NCT05099744).

### 2.2. Participants

The participants met the following inclusion criteria: (i) age over 18 years and (ii) clinical stress levels determined by a predefined cut-off point. Specifically, patients were included if they scored above the 80th percentile on the Perceived Stress Scale (PSS-10) [47], i.e., reported distress scores higher than 22 for males and 20 for females. The exclusion criteria were as follows: (i) use of a defibrillator or pacemaker, (ii) pregnancy, and (iii) receiving psychological support or therapy during the intervention.

The study was promoted within the university through institutional email and the university counseling service’s website, as well as through social networks.

Individuals who expressed interest in participating completed a brief screening questionnaire to determine whether they met the inclusion criteria. Data collection took place in the physiological laboratory of a major university in northern Portugal between March 2022 and January 2025.

### 2.3. Interventions

All participants, regardless of the group to which they were allocated, received 12 individual sessions, each lasting 50 min, conducted twice a week with a minimum interval of 24 h between sessions.

The relaxation intervention was carried out by three certified and trained clinical psychologists following a written protocol based on Schultz’s autogenic relaxation training [31]. The sessions were conducted in a quiet room with subdued light and soft background music. The participants sat comfortably while a certified clinical psychologist guided them through Shultz’s AT or FSM.

The TimeWaver Frequency system was used to apply FSM therapy. This device, designed for administering microcurrent frequency (TimeWaver Home GmbH, Märkisch Linden, Germany), is approved and certified under the European Directive 93/42/EWG class IIa, making it valid for use across Europe. This system combines hardware and software, operated by the researcher using a laptop computer. The device allows for continuous analysis and transmission of the appropriate microcurrent frequencies using the two electrodes. The system delivers low-intensity, sub-sensory electrical currents through hand electrodes while participants are seated in a relaxing position. The participants were informed that they could perceive a tingling sensation or nothing at all during the session. In this study, an adaptation of the therapy protocol for anxiety reduction was used to fit the 50-min intervention. Step 1 comprised a frequency range of 1–1000 Hz with a fixed amplitude of 7 volts, a duration per frequency of 10 s, and a fixed rectangular wave [48]. This type of wave has an inflexible nature, contrary to sinusoidal waves, keeping the same amplitude and form, which is ideal for enhancing specific cellular responses to break dysfunctional patterns [49]. The total duration of this procedure was 9 min. Step 2 used the indications feature to analyze the frequencies chosen by the software to analyze, in real-time, with a threshold of 62%, the two most relevant positive and negative frequencies, and the single most relevant positive and negative anxiety frequencies. The 62% threshold was selected based on an optimization of sensitivity and specificity, allowing for effective discrimination between functional and dysfunctional states without compromising specificity. Several studies adopted a similar threshold, which reflects the inflection point at which the effects of a disturbance are clinically relevant and physiologically measurable [50]. Relevant negative frequencies are associated with physiological neural hyperactivity, and the two relevant positive frequencies are associated with neural regulation and sedation to restore balance and emotional stabilization. We chose the two most relevant frequencies to potentiate the therapeutic effects and decrease the dispersion with other frequencies. The same applies to anxiety frequencies, which function as a modulator of emotional and physiological factors to favor the body’s adaptive response. The procedure took 30 min. Step 3 repeated step 1 for another 9 min. Therefore, each participant received 9 min + 30 min + 9 min of FSM therapy, in this sequence, to keep the intervention within the 50 min session.

#### 2.3.1. Experimental Group 1 (EG1)

The EG1 participants received 12 FSM sessions. The participants sat while holding two electrodes in their hands.

#### 2.3.2. Experimental Group 2 (EG2)

The EG2 participants received 6 FSM sessions, followed by 6 sessions of relaxation.

#### 2.3.3. Active Control Group (ACG)

Participants in the ACG group received 12 sessions of relaxation.

#### 2.3.4. Passive Control Group (PCG)

Participants in the PCG group received 6 placebo FSM sessions, i.e., the device was turned on (green light was visible) while the participant was holding the electrodes, but no frequencies were being delivered, followed by 6 sessions of relaxation. A detailed overview of the session structure for each group is presented in Table 1.

### 2.4. Outcomes

A sociodemographic questionnaire was developed for this study to assess sociodemographic (e.g., age, gender, education level) and clinical variables (e.g., use of alcohol and tobacco consumption, use of medications).

#### 2.4.1. Primary Outcome Measures

The **Patient Health Questionnaire**-15 (PHQ-15) [51] evaluates somatic symptoms through 15 items distributed across three subscales: general pain and discomfort (9 items), sexual and cardiac discomfort (4 items), and state of dizziness (2 items). Each item is scored on a three-point Likert scale, ranging from 0 (“not bothered at all”) to 2 (“bothered a lot”). Higher scores indicate more somatic symptoms. Cronbach’s alpha for the full scale was 0.88 and 0.78 in the Portuguese version and in this study, respectively.

The **Satisfaction with Life Scale** (SWLS) [52] is comprised of 5 items that assess an individual’s overall perception of their satisfaction with life. Each item is scored on a five-point Likert scale, ranging from 1 (“strongly disagree”) to 5 (“strongly agree”). Higher scores indicate greater satisfaction with life. In the Portuguese version, Cronbach’s alpha was 0.77, and in the present study, it was 0.86.

#### 2.4.2. Secondary Outcome Measures

The **Perceived Stress Scale** (PSS-10) [47] includes 10 items that assess perceived stress regarding life events over the previous month. Each item is scored on a five-point Likert scale, from 0 (“never”) to 4 (“very often”). Higher scores indicate higher levels of perceived stress. According to Trigo et al. [47], the cut-off point for clinically significant stress levels is 20 for men and 22 for women (80th percentile). In the Portuguese version, Cronbach’s alpha was 0.87, and 0.88, in the present study.

The **Hospital Anxiety and Depression Scale** (HADS) [53] includes 7 items to assess depression and 7 items to assess anxiety using a four-point Likert scale. The total score ranges from 0 to 42, with higher scores indicating greater emotional distress. In the Portuguese version, Cronbach’s alpha was 0.76 for anxiety and 0.81 for depression. This study used the emotional distress (total) score, presenting a Cronbach’s alpha of 0.84.

The **Difficulties in Emotion Regulation Scale** (DERS) [54] assesses the difficulties in emotion regulation and consists of 36 items divided into six subscales: non-acceptance of negative emotions (6 items), inability to engage in goal-directed behavior when experiencing negative emotions (5 items), difficulties in controlling impulsive behavior when experiencing negative emotions (6 items), limited access to emotion regulation strategies perceived as effective (8 items), emotional awareness (6 items), and emotional clarity (5 items). Items are scored on a five-point Likert scale, ranging from 1 (“almost never” (0–10%)) to 5 (“almost always” (91–100%)). Higher scores indicate more emotion regulation difficulties. In the Portuguese version, Cronbach’s alphas were ≥0.75 for the subscales and 0.92 for the total scale. This study used the total scale, with a Cronbach’s alpha of 0.94.

The **Affective State Inventory-Reduced** (ASI-R) [55] assesses the intensity of positive and negative emotional states through 19 items divided into five subscales: negative emotions (4 items), euphoric arousal (4 items), self-efficacy (4 items), warmth (4 items), and serenity (3 items). Each item is scored on a five-point Likert scale, ranging from 1 (“very little or not at all”) to 5 (“extremely”). Higher scores indicate greater intensity of emotional states. In the Portuguese version, Cronbach’s alphas ranged from 0.76 to 0.90. In this study, the alphas for the subscales were: 0.85 for negative emotions, 0.76 for euphoric arousal, 0.87 for self-efficacy, 0.79 for warmth, and 0.90 for serenity.

### 2.5. Sample Size

Although a formal sample size calculation was not required for the pilot study, due to the lack of previous studies to inform this calculation, an estimation of the sample size was made based on the recommendations by Cocks and Torgerson [56]. Assuming an average effect size of 0.50, a statistical power of 0.80, and a statistical significance level of 0.05, a total of 48 participants were required, i.e., 12 participants per arm. However, we were able to enroll 58 participants, which provided a slightly larger dataset for estimating the key parameters and trends. While such an increase was unlikely to substantially change the outcomes of a pilot study, it did enhance the reliability of preliminary estimates and supported the design of a future powered trial.

### 2.6. Randomization

Randomization was conducted using an online random number generator (https://www.graphpad.com/quickcalcs/ [accessed on 1 December 2021]) by an independent researcher external to the study to ensure allocation concealment. The participants were blinded to their assigned group. Each participant was allocated to one of the four possible groups following a sequential method (EG1, EG2, ACG, PCG).

### 2.7. Ethical Considerations

The participants voluntarily enrolled in the study and signed an informed consent form. This study was approved by the Ethics Committee for Research in Social and Human Sciences of the Ethics Council of the University of Minho (CEICSH 013/2019). All study procedures followed the guidelines of the Declaration of Helsinki (1989) of the World Medical Association, and the study did not involve any risk or harm to the participants.

### 2.8. Data Analysis

Descriptive statistics were performed using IBM SPSS Statistics software, version 29. Linear mixed models (LMMs) were employed to analyze both within-group and between-group changes in outcome variables. LMMs are a powerful statistical approach for analyzing data with repeated measures on the same individuals or clustered observations within groups. In the present study, the participants were assessed over time, with different numbers of observations across individuals. Unlike repeated-measures ANOVA, LMMs can efficiently handle unbalanced designs by using all available data. The model included time (T0/T1/T2), treatment group (EG1/EG2/ACG/PCG), and the interaction between group and time as fixed effects. The effects of intervention groups regarding the outcomes measured at T0, T1, and T2 were examined, controlling for physical exercise. An estimate of effect size and the 95% confidence intervals for each predictor variable were computed, comparable to Cohen’s d [57], in the context of linear mixed models [58]. Since physical exercise is associated with a better ability to regulate negative emotions [59] and less perceived psychological stress [60], physical exercise was controlled for in all analyses.

Statistical analyses were performed using the R statistical computing environment (version 3.6.2, R Core Team, Vienna, Austria). The *lme4* and *ggplot2* packages were used for statistical analysis and data visualization. There were no missing values in the predictor variables; *p*-values below 0.05 were considered statistically significant.

## 3. Results

### 3.1. Participant Recruitment

**Figure 1:**
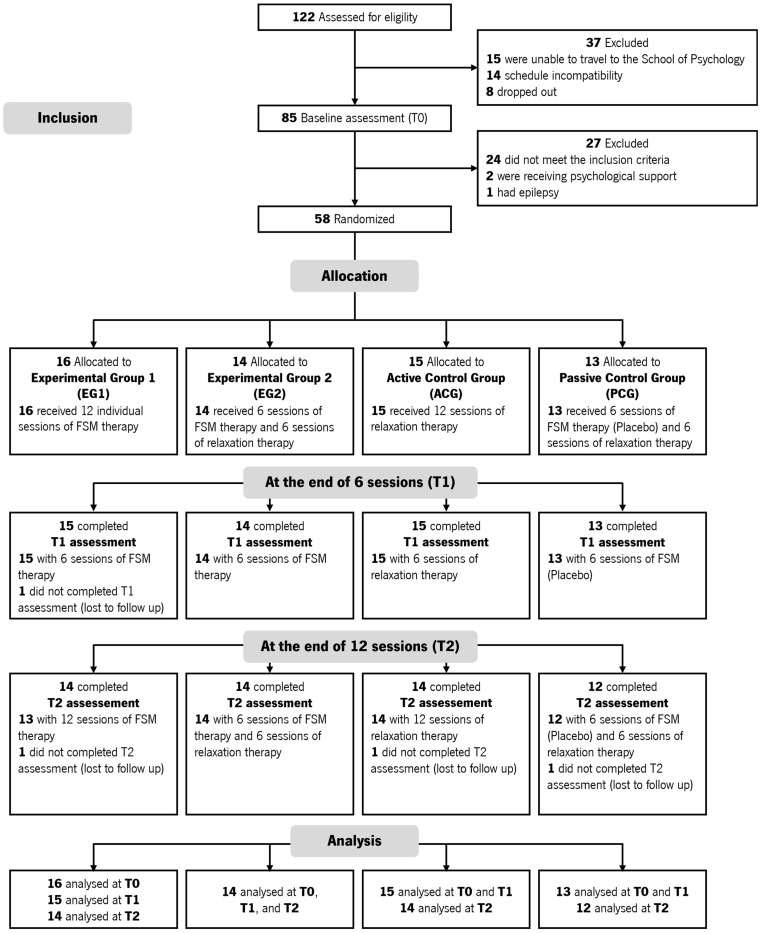
CONSORT flow diagram.

One hundred and twenty-two participants were assessed for eligibility, but only 85 completed the baseline assessment (Figure 1). From those, only 58 randomized participants were assigned to the groups, i.e., 68.24% of the participants who were assessed at baseline. Therefore, the rate of inclusion in the study was 44.7%. As a result, 16 participants were allocated to EG1; 14 participants to EG2; 15 participants to ACG; and 13 participants to PCG (Figure 1). Thus, a total of 58 participants, with an average age of 25.29 years (*SD* = 10.62), completed the study protocol.

### 3.2. Baseline Data

No differences were found between the groups at T0 in any of the variables. Table 2 shows the sociodemographic and clinical data of the participants by group.

### 3.3. Outcomes and Estimation

Table 3 shows the scores of the primary and secondary outcomes by group at the three assessment moments.

### 3.4. Differences Between Groups over Time

#### 3.4.1. Primary Outcomes

**Figure 2:**
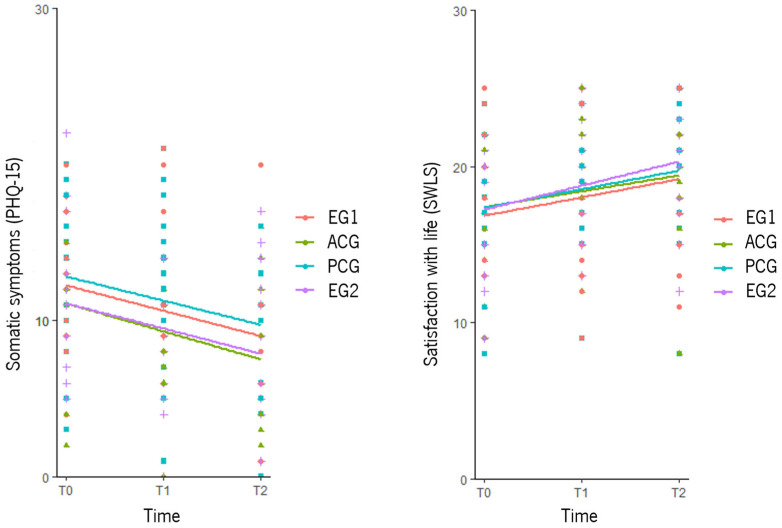
Graphical representation of the between- and within-group differences in the primary outcomes. T0 = baseline assessment; T1 = after the sixth session; T2 = after the 12th session; EG1 = experimental group 1; EG2 = experimental group 2; ACG = active control group; PCG = passive control group; PHQ-15 = Patient Health Questionnaire-15; SWLS = Satisfaction with Life Scale.

Participants in the ACG reported more somatic symptoms than those from EG2 (B = 5.005, *p* < 0.05) at T1. Satisfaction with life showed no significant differences (Table 4). Figure 2 shows changes (between and within groups) in somatic symptoms and satisfaction with life over time for the four groups.

#### 3.4.2. Secondary Outcomes

**Figure 3:**
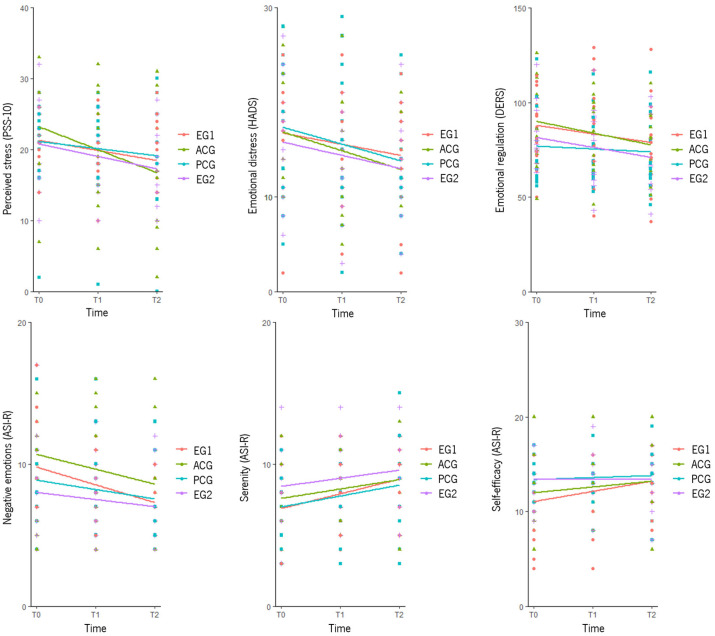
Graphical representation of the between- and within-group differences in the secondary outcomes. T0 = baseline assessment; T1 = after the sixth session; T2 = after the 12th session; EG1 = experimental group 1; EG2 = experimental group 2; ACG = active control group; PCG = passive control group; PSS-10 = Perceived Stress Scale; HADS = Hospital Anxiety and Depression Scale; DERS = Difficulties in Emotion Regulation Scale; ASI-R = Affective State Inventory—Reduced (subscales).

Participants in the ACG reported higher negative emotions than those from EG2 (B = 2.581, *p* < 0.05) at T1. There were no significant differences for emotional distress, emotion regulation, perceived stress, euphoric arousal, self-efficacy, warmth, and serenity (Table 4). Figure 3 shows changes (between and within groups) in perceived stress, emotional distress, emotion regulation, and emotional states over time for the four groups. Only variables with significant results were included.

### 3.5. Differences Within Groups over Time

#### 3.5.1. Primary Outcomes

Somatic symptoms significantly decreased in EG1 from T0 to T2 (B = −3.582, *p* < 0.01); in EG2, from T0 to T1 (B = −4.786, *p* < 0.01) and from T0 to T2 (B = −3.214, *p* < 0.05); in the ACG, from T0 to T2 (B = −2.803, *p* < 0.05) and from T1 to T2 (B = −2.870, *p* < 0.01); and in the PCG, from T0 to T2 (B = −3.087, *p* < 0.05).

Satisfaction with life significantly increased in EG1 from T0 to T1 (B = 1.857, *p* < 0.01) and from T0 to T2 (B = 1.915, *p* < 0.05); in EG2, from T0 to T1 (B = 2.214, *p* < 0.05) and from T0 to T2 (B = 3.071, *p* < 0.05); in the ACG, from T0 to T2 (B = 2.032, *p* < 0.01) and from T1 to T2 (B = 1.966, *p* < 0.001); and in the PCG, from T1 to T2 (B = 1.376, *p* < 0.05) (see Table 4 and Figure 2).

#### 3.5.2. Secondary Outcomes

Perceived stress significantly decreased in EG1 from T0 to T1 (B = −2.859, *p* < 0.05); in EG2, from T0 to T1 (B = −3.500, *p* < 0.05) and from T0 to T2 (B = −3.500, *p* < 0.05); and in the ACG, from T0 to T2 (B = −5.742, *p* < 0.001) and from T1 to T2 (B = −3.476, *p* < 0.05). No significant differences were found in the PCG.

Emotional distress significantly decreased in EG1 from T0 to T1 (B = −3.167, *p* < 0.05) and from T0 to T2 (B = −2.958, *p* < 0.05); in EG2, from T0 to T1 (B = −2.500, *p* < 0.05) and from T0 to T2 (B = −1.039, *p* < 0.05); in the ACG, from T0 to T2 (B = −2.775, *p* < 0.05) and from T1 to T2 (B = −1.842, *p* < 0.05); and in the PCG, from T0 to T2 (B = −2.651, *p* < 0.05) and from T1 to T2 (B = −2.294, *p* < 0.05).

Difficulties in emotion regulation significantly decreased in EG1 from T0 to T2 (B = −10.738, *p* < 0.05) and from T1 to T2 (B = −6.750, *p* < 0.05); in EG2, from T0 to T2 (B = −10.429, *p* < 0.05); and in the ACG, from T0 to T2 (B = −6.988, *p* < 0.05) and from T1 to T2 (B = −5.988, *p* < 0.05). No significant differences were found in the PCG.

Regarding emotional states, negative emotions significantly decreased in EG1 from T0 to T1 (B = −1.534, *p* < 0.05) and from T0 to T2 (B = −2.043, *p* < 0.05); in the ACG, from T0 to T2 (B = −1.857, *p* < 0.05) and from T1 to T2 (B = −1.657, *p* < 0.05); and in the PCG, from T1 to T2 (B = −1.691, *p* < 0.05). No significant differences were found in EG2. Self-efficacy significantly increased in EG1 from T0 to T2 (B = 1.746, *p* < 0.01) and from T1 to T2 (B = 0.972, *p* < 0.05), but no significant differences were found in EG2, the ACG, and the PCG. Serenity also showed no significant differences in the PCG and EG2. However, serenity significantly increased in EG1 from T0 to T2 (B = 1.322, *p* < 0.05) and in the ACG from T0 to T1 (B = 1.267, *p* < 0.05) and from T0 to T2 (B = 0.628, *p* < 0.05). No significant differences were found regarding the euphoric arousal and warmth subscales (Table 4 and Figure 3).

## 4. Discussion

This pilot RCT assessed the effectiveness of FSM therapy alone (EG1) and combined with the relaxation intervention (EG2), compared to the relaxation intervention alone (ACG) and combined with placebo (PCG). Between-group analysis showed that the ACG participants reported more somatic symptoms than those from EG2 at T1, suggesting that FSM was more effective in reducing somatic symptoms than relaxation after six sessions. Previous research on somatic disorders (e.g., chronic pain) has already suggested that FSM may produce immediate positive effects after each session [33]. A recent review corroborated these findings by showing that FSM enhances physiological activity, with individuals who received FSM reporting better health outcomes (e.g., less pain) in comparison to the control groups [44]. Furthermore, the fact that FSM was effective in somatic symptoms after only six sessions may be important for informing a (future) definitive RCT targeting somatic complaints of stress.

The ACG participants reported more negative emotions than those from EG2 at T1, indicating that six sessions of FSM were more effective in reducing negative emotions compared to the relaxation intervention. Although there is a lack of studies comparing the efficacy of FSM with relaxation interventions, regarding the reduction in negative emotions, some studies using FSM have shown promise in alleviating symptoms associated with negative emotions (e.g., [45]). As suggested by the previous findings, FSM seems to offer faster and more profound relief from negative affect, highlighting its potential advantage in mitigating negative emotions. However, further research is warranted to directly compare its efficacy with AT relaxation intervention.

According to within-group comparisons, somatic symptoms significantly decreased in all the groups from T0 to T2, from T0 to T1 in EG2 (after six sessions of FSM), and from T1 to T2 in the ACG (i.e., continued to decline after six to 12 sessions- of relaxation). These results suggest that six to 12 sessions of FSM or relaxation were sufficient to reduce the burden of somatic complaints in individuals with clinical stress levels, which is consistent with previous studies [29,45]. In fact, in addition to FSM, relaxation interventions have already been shown to significantly improve conditions associated with chronic somatic diseases and related mental health disorders [29,30].

Satisfaction with life improved in EG1 and EG2 from T0 to T1 and from T0 to T2, indicating that both interventions produced positive effects, which were maintained and amplified over time. The early and sustained improvement observed in EG1 and EG2 suggests that FSM had a more immediate and lasting effect compared to relaxation. FSM is believed to influence both physical and emotional well-being, which are the key determinants of satisfaction with life [61]. FSM has shown promise in addressing emotional distress [32], psychological morbidity (e.g., anxiety and depression) [41], sleep disorders [36], inflammation [37], and chronic pain (e.g., [38]), promoting physical recovery. Given that physical discomfort and emotional distress are known to negatively impact satisfaction with life, it is plausible that FSM contributes to improvements in individuals’ subjective well-being. Additionally, there were no differences in satisfaction with life in the PCG from T0 to T1 when the participants received only placebo FSM, suggesting that the effects observed in both experimental groups were attributable to FSM rather than to participants’ expectations. Finally, the significant improvements observed from T0 to T2 in the ACG, as well as from T1 to T2 in both the ACG and the PCG, indicate that relaxation had a cumulative effect over time, resulting in continuous improvements after the sixth and twelfth sessions. The sustained benefits of relaxation on satisfaction with life reveal that this intervention may contribute to positive cognitive evaluations of life, possibly through its ability to alleviate stress and enhance emotional well-being [27].

The analysis of the secondary outcomes revealed that perceived stress significantly decreased from T0 to T2 in relaxation (EG2 and the ACG), suggesting the cumulative effect of relaxation on stress reduction, which is consistent with previous findings. A study by Litwic-Kaminska et al. [62] showed that relaxation plays an important role in stress regulation and adjustment in several functional disorders (e.g., trauma) by encouraging the body’s ability to restore balance and promoting overall mental and physical well-being. Interestingly, the EG1 participants showed a decrease in perceived stress after the first six sessions of FSM, indicating that six sessions of FSM were enough to reduce perceived stress. This finding is in accordance with studies showing that FSM therapy can foster rapid physiological changes, such as reduced muscle tension, lower cortisol levels, and improved autonomic nervous system balance [63]. The early improvements in stress noted in EG1 could be attributed to the intervention’s ability to influence the autonomic nervous system, leading to a decrease in sympathetic nervous system activity, which is typically elevated in stressful situations. Additionally, the EG2 participants reported a significant reduction in perceived stress from T0 to T1 (after six sessions of FSM) and from T0 to T2 (after six sessions of FSM and six sessions of relaxation), suggesting that both FSM alone and FSM combined with relaxation interventions are effective. The synergistic effect of combining both treatments could have enhanced the overall impact, leading to a more substantial and sustained stress reduction compared to either treatment alone. The lack of perceived stress reduction in the PCG contrasts with the effects observed in EG2 and the ACG, where relaxation led to significant improvements. Therefore, we may hypothesize that six sessions of relaxation may not be sufficient to decrease perceived stress.

Difficulties in emotion regulation significantly decreased in both experimental groups, as expected. However, EG1 and EG2 showed no differences from T0 to T1, while the participants received FSM, which may indicate that complex cognitive and emotional processes, such as emotion regulation, require more than six sessions of FSM to elicit meaningful therapeutic changes. Future studies are needed to test this hypothesis. Similarly, the ACG exhibited a significant decrease from T0 to T2 and from T1 to T2, further supporting the positive effect of relaxation on emotion regulation, which is consistent with previous research on self-induced relaxation techniques, in particular, AT [27,64]. Further research with larger samples should examine specific difficulties in the emotion regulation process (e.g., non-acceptance of negative emotions, emotional awareness, and emotional clarity) to provide deeper insights into which facets of emotion regulation are most responsive to these therapeutic modalities and inform the development of more targeted intervention programs.

Regarding emotional states, both treatments had a positive effect on negative emotions and serenity, indicating that FSM and relaxation interventions contributed to a decrease in negative emotions and an increase in serenity. The PCG participants reported a reduction in negative emotions only from T1 to T2, supporting the effectiveness of relaxation in alleviating negative emotions. This finding aligns with prior research documenting the beneficial effects of relaxation on both negative emotions and serenity [25,26,29]. Self-efficacy improved only after six to 12 sessions of FSM. The potential of FSM therapy to modulate the neural pathways linked to interoception and emotion regulation may help to understand these results (e.g., [45]). In sharp contrast, Stößlein and Kuypers [43] found no effects of FSM on emotional states; therefore, future studies should examine the impact of FSM therapy using specific frequencies targeted at emotional functioning to shed light on these results.

Euphoric arousal and warmth emotions showed no significant differences, which may indicate that FSM and relaxation treatments were not effective in promoting positive emotions such as excitement and warmth. Some studies (e.g., [45]) suggest that FSM and relaxation can help regulate stress and anxiety, but may not necessarily trigger the emotional peaks associated with more intense positive emotions, since these emotions often require more active engagement with external stimuli or personal achievements. The positive effects of treatments on self-efficacy and serenity suggest that FSM and relaxation may be valuable in managing patients’ emotional states, particularly those with high stress levels. Nonetheless, the lack of comparable outcomes in EG2 warrants further studies to identify potential barriers and explore strategies to enhance the effectiveness of the intervention.

Significant improvements were observed in emotional distress in all the groups from T0 to T2. Particularly, FSM was shown to have a positive effect after six or 12 sessions (EG1 and EG2), while relaxation was effective only after six to 12 sessions (ACG), or after six sessions in combination with FSM (EG2). Similar to negative emotions, the PCG participants reported significant reductions in emotional distress only after receiving the relaxation sessions, but not while receiving placebo FSM, supporting the efficacy of six sessions of relaxation in reducing emotional distress. However, no significant changes were observed in perceived stress, emotion regulation, self-efficacy, or serenity after six sessions of relaxation (PCG). One may hypothesize that more complex cognitive–emotional processes need more intervention time, as found in other studies [65].

Overall, within-group analysis suggests that relaxation may serve as a reliable coping strategy for distressed individuals, helping them deal with potential affective and mood dysregulation associated with life stressors and/or preexisting emotional vulnerabilities, thereby mitigating the negative emotional burden experienced by these individuals. In this regard, the EG1 participants reported similar results for emotional distress, emotion regulation difficulties, negative emotions, self-efficacy, and serenity. Noteworthily, FSM showed earlier effects (T0 to T1) in perceived stress, emotional distress, and negative emotions, while six sessions of relaxation showed an effect only on serenity. Thus, FSM seems to have a more immediate effect in promoting adaptive emotional functioning, with sustained improvements in T2 suggesting the long-term benefits of this intervention. Although both interventions showed significant and positive changes at different assessment moments, FSM seems to have a greater range in several dimensions of emotional functioning.

Compared to traditional relaxation techniques, the present findings indicate that the FSM therapy produced more rapid effects, as evidenced by earlier improvements in perceived stress and negative affect. The results align with studies indicating that FSM therapy modulates autonomic nervous system balance by reducing sympathetic activation and enhancing the parasympathetic tone [63], thereby promoting recovery processes through improved autonomic regulation. These physiological effects are believed to result from frequency-specific stimulation of pathways involved in tissue repair and neural regulation [37].

While long-term outcomes were not evaluated in the current study, the prior literature suggested that the benefits of FSM therapy may persist over time, depending on treatment frequency, duration, and individual response [45]. Although the findings from this study, regarding the efficacy of FSM therapy over 12 weeks, are promising, further longitudinal research is needed to examine the sustainability of FSM’s therapeutic effects, especially concerning mental health outcomes.

### Limitations and Future Studies

The present study has limitations that must be acknowledged, such as the small sample size. Blinding in this study was not feasible; nonetheless, the findings suggest the potential feasibility of implementing novel interventions such as FSM for distress and emotion regulation. In addition, critical contextual factors should be considered before initiating an intervention for somatic symptoms, which need to be stratified by all the groups, which was not performed in the present study.

In this study, all the participants benefited from one of the interventions presented in the consent form, as required by the university’s ethics committee, and, therefore, the PCG group received only six placebo FSM sessions instead of 12. The reliance on self-reported measures of somatic symptoms, stress, and emotion regulation is also a limitation. Future studies should incorporate objective physiological indicators (e.g., cortisol levels, heart rate variability) to provide more robust evidence of the interventions’ effects.

An efficacy RCT also needs to account for the number of sessions, as findings from this pilot study suggest that cognitive–emotional processes, such as emotion regulation, may require 12 sessions, whereas physiological responses to perceived stress appear to be effectively addressed within six sessions. Finally, future RCTs should include extended follow-up periods to assess the long-term effects of FSM therapy and relaxation interventions.

## 5. Conclusions

FSM is a relatively new technology that has shown promising results in decreasing emotional distress and difficulties in emotion regulation. Compared to relaxation, FSM was more efficient in decreasing somatic symptoms and negative emotions. This study raised several questions regarding the number of sessions required for more complex cognitive and emotional processes, such as emotion regulation or satisfaction with life. A future definitive long-term RCT with a larger sample is needed to clarify the effects of FSM therapy on mental health outcomes. FSM, either alone or combined with relaxation, may help to address the increasing emotional and physical health challenges faced by individuals in today’s world.

## Figures and Tables

**Table 1:** Structure of sessions per group.

Session	EG1: FSM Only	EG2: FSM + Relaxation	ACG: Relaxation Only	PCG: Placebo FSM + Relaxation
1–6	FSM	FSM	Relaxation	Placebo FSM
7–12	FSM	Relaxation	Relaxation	Relaxation
Total	12 FSM sessions	6 FSM + 6 relaxation sessions	12 relaxation sessions	6 placebo FSM + 6 relaxation sessions

Note: EG1 = experimental group 1; EG2 = experimental group 2; ACG = active control group; PCG = passive control group; FSM = frequency-specific microcurrent; placebo FSM = electrodes held with the device on, but no frequency delivered.

**Table 2:** Sociodemographic and clinical characterization of the participants by group at baseline.

		Baseline (T0)				
		EG1 (*n* = 16)	EG2 (*n* = 14)	ACG (*n* = 15)	PCG (*n* = 13)	Total (*n* = 58)
Categorical Variables		*n* (%)	*n* (%)	*n* (%)	*n* (%)	*n* (%)
Gender						
Men		5 (31.3)	1 (7.1)	1 (6.7)	2 (15.4)	9 (15.5)
Women		11 (68.8)	13 (92.9)	14 (93.3)	11 (84.6)	49 (84.5)
Marital status						
Single		13 (81.3)	14 (100)	14 (93.3)	11 (84.6)	52 (89.7)
Married		1 (6.3)	0 (0)	1 (6.7)	1 (7.7)	3 (5.2)
Living with a partner		1 (6.3)	0 (0)	0 (0)	0 (0)	1 (1.7)
Divorced or separated		1 (6.3)	0 (0)	0 (0)	1 (7.7)	2 (3.4)
University student						
No		2 (12.5)	0 (0)	2 (13.3)	2 (15.4)	6 (10.3)
Yes		14 (87.5)	14 (100)	13 (86.7)	11 (84.6)	52 (89.7)
Physical activity						
No		6 (37.5)	4 (28.6)	7 (46.7)	6 (46.2)	23 (39.7)
Yes		10 (62.5)	10 (71.4)	8 (53.3)	7 (53.8)	35 (60.3)
Tobacco consumption						
No		15 (93.8)	14 (100)	14 (93.3)	12 (92.3)	55 (94.8)
Yes		1 (6.3)	0 (0)	1 (6.7)	1 (7.7)	3 (5.2)
Alcohol consumption						
No		7 (43.8)	8 (57.1)	4 (26.7)	8 (61.5)	27 (46.6)
Yes		9 (56.3)	6 (42.9)	11 (73.3)	5 (38.5)	31 (53.4)
Medication ^a^						
No		8 (50)	13 (92.9)	13 (86.7)	11 (84.6)	45 (77.6)
Yes		8 (50)	1 (7.1)	2 (13.3)	2 (15.4)	13 (22.4)
**Continuous Variables**	**Min–Max**	**M (*SD*)**	**M (*SD*)**	**M (*SD*)**	**M (*SD*)**	**M (*SD*)**
Age	18–62	28.94 (11.85)	21.50 (5.11)	24.33 (10.90)	26.00 (12.53)	25.29 (10.62)
Educational level (in years)	12–25	15.92 (4.77)	14.33 (2.93)	13.50 (1.45)	13.45 (1.37)	14.35 (3.13)

Note: all the participants were Portuguese. EG1 = experimental group 1; EG2 = experimental group 2; ACG = active control group; PCG = passive control group; % = percentage; Min = minimum; Max = maximum; M = mean; *SD* = standard deviation. ^a^ Only medication that included psychotropic drugs was considered.

**Table 3:** The participants’ psychological characterization by group at T0, T1, and T2.

					T0 (*N* = 58)				T1 (*N* = 57)				T2 (*N* = 54)			
					EG1 (*n* = 16)	EG2 (*n* = 14)	ACG (*n* = 15)	PCG (*n* = 13)	EG1 (*n* = 15)	EG2 (*n* = 14)	ACG (*n* = 15)	PCG (*n* = 13)	EG1 (*n* = 14)	EG2 (*n* = 14)	ACG (*n* = 14)	PCG (*n* = 12)
Continuous Variables	Min–Max				M (*SD*)				M (*SD*)				M (*SD*)			
	EG1	EG2	ACG	PCG												
Emotional distress (HADS)	5–28	6–27	4–29	2–25	17.13 (5.23)	16.14 (6.19)	17.07 (7.09)	16.46 (5.98)	13.67 (5.70)	13.64 (4.88)	16.13 (7.61)	16.08 (5.80)	13.14 (3.94)	13.36 (5.24)	13.50 (6.11)	14.08 (6.24)
Perceived stress (PSS-10)	8–28	10–32	2–33	0–30	21.44 (3.71)	21.36 (5.87)	23.40 (6.12)	21.23 (6.86)	18.00 (4.78)	17.86 (4.74)	21.13 (7.19)	19.85 (6.97)	18.64 (4.88)	17.86 (5.04)	17.29 (8.88)	19.25 (8.07)
Emotion regulation (DERS)	46–135	54–120	37–129	46–123	90.50 (23.89)	81.57 (15.49)	86.67 (17.92)	76.77 (20.84)	85.80 (19.99)	75.86 (20.32)	85.67 (25.00)	75.46 (18.72)	77.93 (19.65)	71.14 (19.60)	77.50 (23.72)	73.75 (21.59)
Negative emotions (ASI-R NE)	4–17	4–17	4–16	4–16	9.81 (2.88)	8.00 (3.44)	10.40 (3.60)	8.46 (2.96)	8.27 (2.37)	7.50 (2.65)	10.20 (4.07)	8.69 (3.75)	7.57 (1.56)	7.00 (2.35)	8.29 (3.38)	7.42 (3.18)
Euphoric arousal (ASI-R EA)	4–15	4–11	4–14	4–13	8.38 (3.10)	7.50 (2.38)	7.60 (2.59)	8.23 (2.28)	8.87 (2.77)	8.43 (2.82)	8.07 (2.76)	8.77 (3.09)	9.29 (2.92)	8.14 (2.69)	8.79 (2.81)	8.58 (2.91)
Self-efficacy (ASI-R SE)	4–17	7–19	6–20	7–19	11.25 (3.47)	13.21 (2.42)	12.07 (3.62)	13.62 (2.02)	12.00 (3.30)	13.79 (2.58)	12.40 (3.33)	13.62 (2.40)	13.36 (2.74)	13.21 (2.61)	13.29 (3.69)	13.67 (3.14)
Warmth (ASI-R W)	5–19	10–20	7–20	7–18	12.94 (3.64)	14.86 (3.46)	13.67 (3.02)	14.62 (1.94)	13.60 (2.64)	14.79 (2.05)	14.20 (2.46)	13.69 (2.50)	14.07 (3.58)	15.00 (2.18)	14.21 (3.04)	15.08 (2.71)
Serenity (ASI-R S)	3–13	3–14	3–12	3–15	7.50 (2.58)	8.43 (3.41)	6.80 (2.65)	6.85 (2.64)	8.53 (1.96)	9.00 (2.29)	8.07 (2.76)	7.77 (2.62)	8.79 (2.81)	9.57 (2.65)	8.86 (2.14)	8.25 (3.33)
Somatic symptoms (PHQ-15)	0–16	4–22	0–21	0–21	11.06 (4.74)	12.14 (5.76)	12.27 (5.35)	12.46 (4.56)	8.80 (4.09)	7.36 (3.41)	12.33 (5.85)	10.15 (5.97)	7.21 (5.03)	8.93 (4.76)	9.14 (4.75)	9.25 (4.33)
Satisfaction with life (SWLS)	8–25	9–25	9–25	8–25	17.00 (3.88)	17.00 (4.08)	17.20 (4.41)	17.46 (4.39)	18.79 (3.49)	19.21 (3.75)	17.27 (4.98)	18.31 (4.03)	18.86 (4.29)	20.07 (3.67)	19.57 (4.38)	19.83 (5.10)

Note: T0 = baseline assessment; T1 = after the sixth session; T2 = after the 12th session; EG1 = experimental group 1; EG2 = experimental group 2; ACG = active control group; PCG = passive control group; Min = minimum; Max = maximum; M = mean; *SD* = standard deviation; HADS = Hospital Anxiety and Depression Scale; PSS-10 = Perceived Stress Scale; DERS = Difficulties in Emotion Regulation Scale; ASI-R = Affective State Inventory—Reduced; ASI-R NE = negative emotions subscale; ASI-R EA = euphoric arousal subscale; ASI-R SE = self-efficacy subscale; ASI-R W = warmth subscale; ASI-R S = serenity subscale; PHQ-15 = Patient Health Questionnaire-15; SWLS = Satisfaction with Life Scale.

**Table 4:** Regression coefficient estimates of the linear mixed-effects model.

Response Variable	HADS	PSS-10	DERS	ASI-R NE	ASI-R EA	ASI-R SE	ASI-R W	ASI-R S	PHQ-15	SWLS
Fixed Effects	B (SE)	B (SE)	B (SE)	B (SE)	B (SE)	B (SE)	B (SE)	B (SE)	B (SE)	B (SE)
Intercept	16.363 (1.1697) ***	23.291 (1.806) ***	84.340 (5.793) ***	10.093 (0.851) ***	8.124 (0.764) ***	12.584 (0.858) ***	13.878 (0.796) ***	6.876 (0.755) ***	12.340 (1.395) ***	17.468 (1.225) ***
T1	−0.933 (1.004)	−2.267 (1.165)	−1.000 (2.808)	−0.200 (0.645)	0.467 (0.654)	0.333 (0.599)	0.533 (0.734)	1.267 (0.628) *	0.067 (0.991)	0.067 (0.653)
T2	−2.775 (1.136) *	−5.742 (1.527) ***	−6.988 (2.989) *	−1.857 (0.875) *	1.098 (0.608)	0.949 (0.635)	0.661 (0.735)	1.980 (0.624) **	−2.803 (1.218) *	2.032 (0.751) **
EG1	0.225 (2.187)	−1.759 (2.343)	1.233 (7.435)	−0.533 (1.097)	0.670 (0.985)	−0.946 (1.110)	−0.626 (1.041)	0.663 (0.985)	−1.126 (1.812)	−0.293 (1.597)
PCG	−0.903 (2.225)	−3.122 (2.385)	−10.833 (7.560)	−1.850 (1.134)	0.866 (1.017)	1.399 (1.129)	0.706 (1.058)	0.205 (1.002)	−0.119 (1.844)	0.303 (1.630)
EG2	−0.651 (2.241)	−2.000 (2.401)	−4.193 (7.617)	−2.281 (1.141)	−0.303 (1.023)	0.947 (1.137)	1.108 (1.065)	1.599 (1.008)	−0.512 (1.856)	−0.304 (1.610)
PA	1.507 (1.500)	0.234 (1.540)	4.986 (5.240)	0.657 (0.687)	−1.124 (0.623)	−1.108 (0.747)	−0.453 (0.654)	−0.162 (0.627)	−0.157 (1.185)	−0.575 (1.112)
T1*EG1	−2.234 (1.1.431)	−0.593 (1.663)	−2.987 (4.014)	−1.334 (0.904)	−0.056 (0.922)	0.440 (0.854)	0.303 (1.046)	−0.139 (0.899)	−1.871 (1.409)	1.790 (0.940)
T2*EG1	−0.183 (1.616)	3.100 (2.160)	−3.750 (4.272)	−0.186 (1.214)	0.046 (0.860)	0.796 (0.904)	0.527 (1.048)	−0.658 (0.895)	−0.779 (1.723)	−0.117 (1.081)
T1*PCG	0.576 (1.445)	1.410 (1.676)	0.429 (4.042)	0.414 (0.929)	−0.181 (0.941)	−0.333 (0.863)	−1.176 (1.056)	−0.552 (0.904)	−1.995 (1.427)	0.779 (0.958)
T2*PCG	0.124 (1.635)	3.825 (2.199)	2.204 (4.305)	0.381 (1.261)	−0.501 (0.877)	−0.570 (0.914)	0.148 (1.058)	−0.346 (0.899)	−0.523 (1.901)	0.190 (1.103)
T1*EG2	−1.567 (1.445)	−1.233 (1.676)	−4.714 (4.042)	−0.300 (0.929)	0.462 (0.941)	0.238 (0.863)	−0.605 (1.056)	−0.695 (0.904)	−3.981 (1.586)	2.148 (0.940)
T2*EG2	−0.011 (1.619)	2.242 (2.172)	−3.441 (4.254)	0.857 (1.247)	−0.455 (0.863)	−0.949 (0.906)	−0.518 (1.049)	−0.837 (0.887)	−0.097 (1.878)	1.039 (1.072)

Note: B = unstandardized coefficient; SE = standard error; PA = physical activity; EG1 = experimental group 1; EG2 = experimental group 2; PCG = passive control group; T1 = after the sixth session; T2 = after the 12th session; HADS = Hospital Anxiety and Depression Scale; PSS-10 = Perceived Stress Scale; DERS = Difficulties in Emotion Regulation Scale; ASI-R = Affective State Inventory—Reduced; ASI-R NE = negative emotions subscale; ASI-R EA = euphoric arousal subscale; ASI-R SE = self-efficacy subscale; ASI-R W = warmth subscale; ASI-R S = serenity subscale; PHQ-15 = Patient Health Questionnaire-15; SWLS = Satisfaction with Life Scale; * *p* < 0.05, ** *p* < 0.01, *** *p* < 0.001.

## Data Availability

For ethical and privacy reasons, the data are not publicly available. The data can be made available upon reasonable request to the corresponding author.
